# New occurrence of *Cirolana capricornica* (*Isopoda*: *Cirolanidae*) from *Epinephelus* *chlorostigma* in Suez Governorate, Egypt

**DOI:** 10.14202/vetworld.2024.150-155

**Published:** 2024-01-20

**Authors:** Mohamad Abdulmohsen, Heba I. Abdel-Mawla, Maather M. El-Lamie, Marwa S. Kamel, Sherief M. Abdel-Raheem, Waleed Rizk El-Ghareeb, Ahmed. M. A. Meligy, Eman M. Abouelhassan

**Affiliations:** 1 Department of Public Health, College of Veterinary Medicine, King Faisal University, Hofuf, Saudi Arabia; 2 Suez Canal University, Faculty of Veterinary Medicine, Department of Animal Behavior and Management, Ismailia, Egypt; 3 Department of Fish Diseases, Animal Health Research Institute, Ismailia Branch, Agriculture Research Center, Ismailia, Egypt; 4 Department of Fish Diseases and Management, Faculty of Veterinary Medicine, Suez Canal University, Ismailia, Egypt; 5 Department of Plant Protection, Faculty of Agriculture, Suez Canal University, Ismailia, Egypt; 6 Department of Animal Nutrition and Clinical Nutrition, Faculty of Veterinary Medicine, Assiut University, 71526 Assiut, Egypt; 7 Food Control Department, Faculty of Veterinary Medicine, Zagazig University, Zagazig 44519, Egypt; 8 Department of Clinical Science, Central Lab, College of Veterinary Medicine, King Faisal University, P.O. Box: 400, Hofuf, Al-Ahsa 31982, Saudi Arabia; 9 Department of Physiology, Agricultural Research Center (ARC), Giza, Egypt; 10 Department of Parasitology, Faculty of Veterinary Medicine, Suez Canal University, Ismailia, Egypt

**Keywords:** *Cirolanidae*, clinical picture, *Crustacea*, *Isopoda*, prevalence

## Abstract

**Background and Aim:**

The isopods of the *Crustacea* are noteworthy. All marine, fresh, and brackish waterways at all depths are home to aquatic organisms. This order also includes species that live on land. This study aimed to report a new occurrence of the isopod *Cirolana capricornica* on the operculum, mouth, and body cavities of *Epinephilus chlorostigma* in the Suez Governorate, Egypt.

**Materials and Methods:**

With the help of fishermen, 50 live *E. chlorostigma* (Linnaeus, 1758) were randomly gathered along the Red Sea coast of the Suez Governorate during November and December 2019 for the current investigation. Isopods were isolated from the fish samples and captured using light and electron microscopy for morphological identification.

**Results:**

Some fish were emaciated, and minute white isopods were attached externally to the skin near the gills and mouth cavity, and internally to the mouth cavity. No correlation was observed between body cavity attachment and gross lesions. The prevalence of infestation was 16%.

**Conclusion:**

*C. capricornica* was identified using optical and electron microscopy to analyze the isopod specimens’ morphology. This scavenging isopod species is newly discovered in Egypt.

## Introduction

Major parasitic crustacean groups such as *Copepoda*, *Branchiura*, *Isopoda*, *Amphipoda*, Barnacles, and *Ostracoda* are well-known. Over 73,000 species and a wide range of parasitic forms make up the class *Crustacea* [1]. Unfortunately, the life cycles of most *Crustacea* are not well understood. All marine, freshwater, and brackish waters contain isopods, which are important members of *Crustacea* that can be found at all depths. This order also includes terrestrial species [2]. Some free-living isopod species have the potential to become parasites, while others are naturally parasitic. Parasitic forms may appear outside the body surface, fins, mouths, gill chambers, and even on the flesh of hosts [3].

Cirolanid isopods are predators or scavengers of surface-dwelling fish and invertebrates. Many parasitize net-caught fish as well as sick or weak fish. Some species are able to strip a fish to the bone in a matter of hours [4]. The distinction between scavengers and predators for some species is still debated [2]. Although the isopods used in the present study were obtained from fish, other scientists disagree that they are real parasites. Gentil-Vasconcelos and Tavares-Dias [5] considered some species of this family, such as “*Excorallana berbadensis*” (Boone, 1918), as parasites of South American freshwater fishes. *Excorallana. tricornis* is considered a facultative parasite of many marine fishes [6], and the family *Cirolanidae* is found in marine, estuarine, and some freshwater environments [7]. *Cirolana* (Leach, 1818) is the most diverse genus, with at least 135 described species [8]. “*Cirolana capricornica*” (Bruce, 1986) belongs to the “Pleonastica group” of *Cirolana* species [4]. This group is distinguished by the presence of pleotelsons with transverse rows of nodule and tubercle ornamentation. Some species of this group have been recorded in East and South Africa [9, 10]. Groupers (Family *Serranidae*, subfamily *Epinephelinae*) includes 15 genera and >159 species. They inhabit tropical and subtropical waters [11], including coral reefs [12]. In the Red Sea and the Gulf of Suez [13], there are also species of high market value. Aggregation behavior during spawning makes it easy to catch them with hooks, lines, and gill nets [14]. Approximately 3708 tons of grouper species were landed in 2016, representing 7.3% of Egyptian Red Sea fish landings [15]. Different parasite species have been reported in dusky grouper populations; for instance, natural outbreaks caused by isopod larvae were observed in both wild and captive *Epinephelus*. On the coast of Libya, dusky groupers have been reported to exhibit skin lesions and dermatitis, which are most likely caused by parasites. In the Adriatic Sea, trematodes firmly attached to the gills, pseudobranchs, and orobranchial chambers of dusky groupers have also been reported by De Benedetto *et al*. [16].

The isopod fauna in the Red Sea is less studied than in other regions of the world. As a result, few cirolanid species are known in the Red Sea. At present, information on this species is scarce, especially in the Red Sea region. The aim of this study was to report a new occurrence of the isopod *C. capricornica* on the operculum, mouth, and body cavities of *Epinephilus chlorostigma* in the Suez Governorate, Egypt.

## Materials and Methods

### Ethical approval

This study was approved by the Ethics Committee of Suez Canal University. All animal experiments were conducted following the guidelines of the Guide for the Care and Use of Laboratory Animals, Faculty of Veterinary Medicine Science, Suez Canal University, Egypt (Approval No. 2022050).

### Study period and location

The study was conducted during November and December 2019 at the Faculty of Veterinary Medicine Science, Suez Canal University, Egypt.

### Sampling and laboratory examination

Fifty live samples of *E. chlorostigma* (Linnaeus, 1748) were collected annually along the coast of the Red Sea with the help of fishermen in an investigation of isopod infestation. During November and December 2019 in the Suez Governorate, the samples were delivered to the Faculty of Veterinary Medicine’s parasitology laboratory at Suez Canal University. The lengths were measured from the snout to the end of the caudal fin by a ruler. Isopod specimens were extracted from eight infected fish and stored immediately in 70% ethanol for examination [17]. Each specimen’s body surface, fins, gills, inner operculum wall, branchial cavity, and buccal cavity were checked for parasitic isopods and identified using the key provided by Bruce [4]. The taxonomically significant structures of the species were illustrated using Adobe Illustrator software (2020).

### Stereo-microscope dissection and analysis

Isopods were isolated and captured using a dissecting stereomicroscope (Olympus Japan SZ40).

### Smear preparations and permanent slides

The isopods were washed, and their contents were evacuated using a ventrally inserted syringe needle. Specimens were then stored in 10% sodium hydroxide for cleaning, dehydrated in serial dilutions of ethyl alcohol (25%, 50%, 75%, and 100%), clarified in xylene, and mounted in Canada balsam [18].

### Scanning electron microscopy

The specimens were cleaned using ultrasonography after being rinsed in tap water. Subsequently, the samples were dehydrated in the ethyl alcohol series, with 1 h between each dilution [19]. Subsequently, the specimens were adhered on SEM stubs by their dorsal or ventral surfaces and dried with liquid carbon dioxide (Blazer Union, F1-9496 Blazer/Furstentun Liechtenstein). Scanning electron microscopy was used to analyze samples coated with gold using a SI5OA sputter coater.

## Results

**Figure-1:**
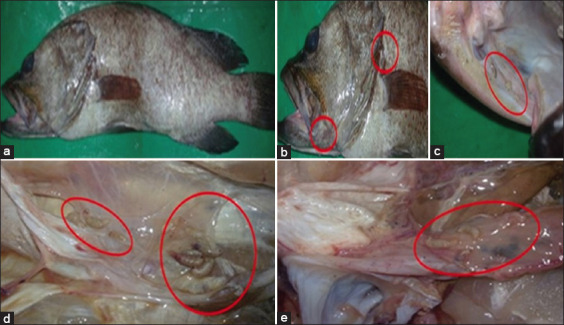
*Epinephilus* *chlorostigma* showing (a) emaciation, *Cirolana capricornica*, (b) attaching near mouth and gill cavities, (c) in the mouth cavity, and (d and e) in the body cavity (Circles).

Fish showed normal appearance and behavior, except for emaciation in the parasitized specimen, and their body weights and lengths ranged from 500 g to 1200 g and 30 cm to 57 cm, respectively (Figure-1a). Small white isopods were attached externally to the skin near the gills and mouth cavities (Figure-1b) and within the mouth cavity (Figure-1c). Internally, isopods were attached to the peritoneum without associated specific gross lesions (Figures-1d and e; Table-1).

**Figure-2:**
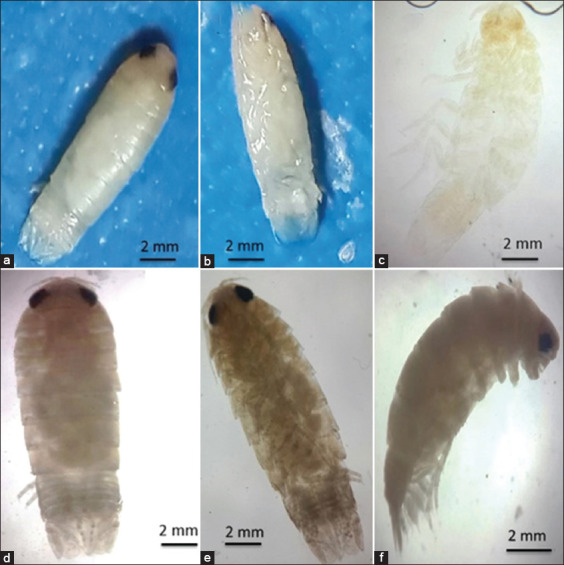
Whole-body images of *Cirolana capricornica*, (a and b) showing their white coloration, (c) Light photomicrograph of permanent preparation of *C. capricornica*, (d) dorsal view, (e) ventral view, and (f) lateral view. Scale bar of a-c, e, and f and please indicated what is host fish species.

**Table-1:** The prevalence and Intensity of *Cirolana capricornica* among the examined fishes.

Host species	No. of fishes examined	No. of fishes infested (% prevalence)	Intensity isopods/fish
*Epinephilus chlorostigma*	50	8 (16)	5–13

### Morphological description

Family *Cirolanidae* Dana, 1852

Genus *Cirolana* Leach, 1818

*C. capricornica* (Bruce, 1986)

**Figure-3:**
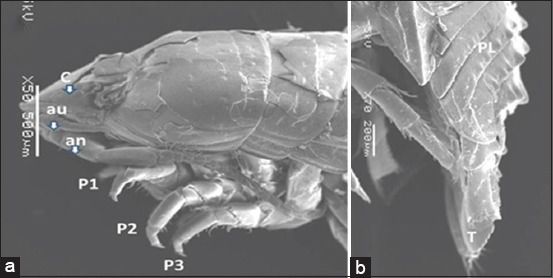
Scanning electron microscope image of *Cirolana capricornica* dorsal view, (a) anterior part and (b) posterior part. au: Antennule, an: Antenna, P=Pereopod, PL=Pleonite, C=Cephalon, T=Telson.

**Figure-4:**
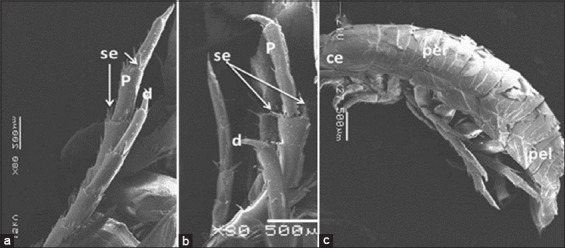
Scanning electron microscope image of *Cirolana capricornica*, (a and b) pereopods, (c) dorsal view. P=Pereopod, per=Pereonite, pel=Pleonite, ce=Cephalon, d=Dactylus, se=Setae.

**Figure-5:**
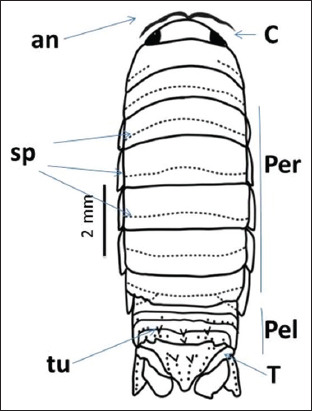
Whole-body illustration of *Cirolana capricornica*. an=Antenna, per=Pereonite, pel=Pleonite, c=Cephalon, T=Telson, sp=Spines, tu=Tubercles.

**Figure-6:**
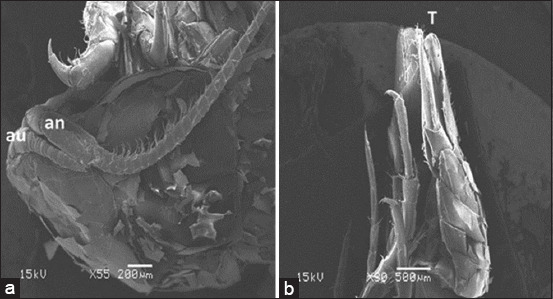
Scanning electron microscope image of *Cirolana capricornica*, ventral view, (a) anterior region and (b) posterior region. au=Antennule, an=Antenna, T=Telson.

The isopod length was 12 mm. Live specimens were white with a faint yellow tinge on the dorsal surface of the pleon segments (Figures-2b, c, d, and e). Cephalon with interocular carina presents on broadly rounded anterior margins (Figures-2a, 2f, and 3a). Posterior margins with impressed transverse lines of all pereonites. Posterolateral denticulate margins of pereonite 7. Coxae on pereonites 2 and 3 were small and not produced, whereas coxae on pereonites 4–7 became progressively more produced (Figure-4). Posterior margins of pleonites 3–5 are denticulate; small and median tubercles are present on both pleonites 4 and 5. Pleotelson is short and fringed with plumose stout spines on the posterior margins; dorsal surfaces bear two rows of tubercles that become progressively smaller posteriorly and scattered small tubercles on anterior lateral surfaces (Figures-1, 3b, and 5). Antennule is short, just reaching pereonite 2; peduncular articles 1 and 2 are short, appearing fused with distinct sutures. The antenna flagellum extends to pereonite 4, composed of approximately 22 articles (Figure-6). Pereopod with 6 tubercles. Pereopods 2 and 3 are similar, less robust, and generally have more and larger spines than pereopods 1. Pereopod 7 with clusters of spines at anterodistal angles to the ischium, merus, and carpus; merus with a spinose distolateral margin; posterior margin of the ischium to the propodus with short marginal spines; and groups of spines at distal angles of the merus and carpus. Pleopods 3–5 with partial exopod suture. Pleopod 1 is a peduncle with hooks and plumose setae on the inner margins, and its spine increases in prominence from pleopods 1–5. Underside of the uropod peduncle armed with two spines, both rami extending beyond the pleotelson. The lateral margin of the endopod is angular, with a small incision near the apex. The posterior margin is broadly rounded, with spines among a fringe of plumose setae. The exopod is narrow, lanceolate, less than half the width of the endopod; the lateral margin is straight with four short spines; the medial margin is fringed with plumose setae and armed with four spines; and the upper lateral surface has five small tubercles (Figures-3b, 6a, and-6b).

## Discussion

Parasitic diseases cause significant fish loss, especially in tropical countries, such as Egypt [20], and different parasitic species have also been reported worldwide, such as in China, East Africa, and Australia [21]. Fish parasites are a critical part of the aquatic ecosystem and are found in natural and cultured fish populations [22]. Little information is available on the ecology of cirolanids, most of which concern only a limited number of isopod species [4]. There is no general cirolanid zone on sand beaches because cirolanids can be present at any level of the beach. Cirolanids are found primarily in crevices, vacant burrows, and cracks in dead coral rocks in coral reefs [23]. The Red Sea is a neglected area with limited studies of isopods, leading many species to remain undescribed [24].

The morphological characteristics of the cirolanid isopod [4] *C. capricornica*, a member of the *Cirolana* “pleonastica” group, conformed to the specimens collected in this study. There are 28 species in this group that have been isolated from different countries all over the world [9]. Some of them have been recorded in Africa [9], and the present infestation is considered the first for this isopod on *Epinephelus* spp.

Cirolanids are usually found attached to fish and have long been considered fish parasites. Bruce indicated that cirolanids are scavengers and predators that attach to fish from which they extract their nutrients and kill confined fishes [25]. Under favorable conditions, *Cirolana fluviatilis* becomes a serious pest and can cause mass mortality in cage-cultured finfish [26]. On the other hand, Keable considered *Natatolana*, one of the cirolanid species, as an opportunistic and voracious scavenger [27] because they usually swarm in large numbers and attack damaged fish, especially at dusk or during the night [28]. Cirolanids also cause severe damage to commercial fishing operations [7] and have been described as feeding on dying fish trapped in trammel nets and long-line fisheries, such as *Notholaena neglecta* reported in Italy [29]. In addition, even cirolanids can attack and kill sharks [6].

To the best of our knowledge, this is the first report of a fish infestation by *C. capricornica* from *E. chlorostigma* in Egypt. The affected fish show emaciation as cirolanids extract nutrients [4]. Blood and tissue from an infested turtle in the gut of *N. neglecta* were also found [2]. *C. capricornica* specimens were found on the external body surface and in the cavity. These parasites were not associated with severe damage to the skin, organs, or tissues. The parasites may have infected the fish only recently, and no obvious damage has yet occurred. Recently, Kirkim isolated *N. neglecta* from *Sparus aurata* (Linnaeus, 1758) (gild-head bream) and *Pagellus* *erythrinus* (Linnaeus, 1758) (common Pandora) with parasites in the eyes and operculum [30]. The latter fish were collected from the southeast Aegean Sea, Turkey. *C*. *fluviatilis* has also been found to cling/feed on the body of moribund and dead cultured *Lates calcarifer* fingerlings from the southwest coast of India. *Cirolana* spp. has also been isolated from the ventral surface of deep-sea shark (*Heptranchias perlo*) from the Bahamas [26]. Finally, isopod scavenging has been reported in the coelomic cavity, on the external surface of organs (liver), and inside the esophagus and skull (salt gland) [2].

## Conclusion

A new occurrence of *C. capricornica* (Cirolanid isopod) on the skin and mouth and in body cavities of *E. chlorostigma* in Suez Governorate, Egypt, should encourage scientists to further study the ecology and biology of this isopod and its effect on wild and cultured marine fish species.

## Authors’ Contributions

MA and HIA: Collected the samples, designed, and supervised the study. MSK and SMA: Data collection and analysis. EMA and MME Supervised the study, conceived the idea, and drafted and edited the manuscript. WRE and AMAM: Collected and analyzed the data and investigated the study. All authors have read, reviewed, and approved the final manuscript.
